# Sensory Symptoms and Signs of Hyperarousal in Individuals with Fragile X Syndrome: Findings from the FORWARD Registry and Database Multisite Study

**DOI:** 10.1007/s10803-023-06135-y

**Published:** 2023-10-16

**Authors:** Ave M. Lachiewicz, Tracy M. Stackhouse, Kristin Burgess, Debra Burgess, Howard F. Andrews, Tse-Hwei Choo, Walter E. Kaufmann, Sharon A. Kidd

**Affiliations:** 1 Department of Pediatrics, Duke University Health System, Durham, NC USA; 2 Developmental FX, Denver, CO USA; 3 North Carolina Fragile X Foundation, Durham, NC USA; 4 Departments of Psychiatry and Biostatistics, Mailman School of Public Health, Columbia University, Irving Medical Center, New York, NY USA; 5 Division of Mental Health Data Science, New York State Psychiatric Institute, New York, NY USA; 6 Department of Human Genetics, Emory University School of Medicine, Atlanta, GA USA; 7 Department of Pediatrics, University of California San Francisco, San Francisco, CA USA

**Keywords:** Fragile X syndrome, Sensory symptoms, Hyperarousal

## Abstract

This study was designed to increase our understanding about characteristics and the impact of sensory symptoms (SS) and signs of hyperarousal (HA) in individuals with fragile X syndrome (FXS) from childhood through early adulthood and by gender. Data derived from the Fragile X Online Registry With Accessible Research Database (FORWARD), a natural history study of FXS, were analyzed using descriptive statistics and multivariate linear and logistic regression models to examine SS and signs of HA, their impact on behavioral regulation and limitations on the subject/family. The sample (N = 933) consisted of 720 males and 213 females. More males were affected with SS (87% vs. 68%) and signs of HA (92% vs. 79%). Subjects who were endorsed as having a strong sensory response had more comorbidities, including behavioral problems. The predominant SS was difficulty with eye gaze that increased with age in both genders. As individuals age, there was less use of non-medication therapies, such as occupational therapy (OT)/physical therapy (PT), but there was more use of psychopharmacological medications and investigational drugs for behaviors. Multiple regression models suggested that endorsing SS and signs of HA was associated with statistically significantly increased ABC-C-I subscale scores and limited participation in everyday activities. This study improves our understanding of SS and signs of HA as well as their impact in FXS. It supports the need for more research regarding these clinical symptoms, especially to understand how they contribute to well-known behavioral concerns.

## Introduction

Fragile X syndrome (FXS) is the most common form of inherited intellectual disability (ID) as well as the most frequent identifiable genetic cause of autism spectrum disorder (ASD), affecting nearly 1/4000–1/7000 males and 1/6000–1/11,000 females in the United States (Sherman et al., 2017). FXS results from a trinucleotide (CGG) repeat expansion in the 5′ untranslated region of the fragile X messenger ribonucleoprotein 1 (*FMR1)* gene on the X chromosome. This expansion, termed full mutation (FM) (> 200 CGG repeats), leads to atypical methylation that results in partial to complete silencing of *FMR1* and, consequently, a marked decrease in the fragile X messenger ribonucleoprotein (FMRP) (Pieretti et al., 1991). FMRP is a RNA-binding protein, which regulates protein synthesis and is critical for brain development and synaptic plasticity (Willemsen et al., 2011).

Since FXS is an X-linked disorder, males are more affected than females with up to 90% having ID in contrast to approximately 25% of females, who present with ID that is milder (Hagerman & Hagerman, 2002; Hagerman et al., 1994; Taylor et al., 1994). The FXS phenotype includes a wide range of physical and neurological abnormalities, such as cranial dysmorphia, strabismus, otitis media, joint hypermobility, and seizures (Hersh et al., 2011; Kidd et al., 2014). The behavioral phenotype includes mild to severe attention-deficit/hyperactivity disorder (ADHD), anxiety, aggressive behaviors, and autistic features or ASD (Bailey et al., 2008; Boyle & Kaufmann, 2010; Kaufmann et al., 2017, 2022). Closely linked to these behavioral problems are sensory symptoms (SS) and hyperarousal (HA). Despite their apparent high prevalence, SS and HA in FXS have not been characterized to the same extent as other behavioral features (Boyle & Kaufmann, 2010). Better understanding of prevalence and impact of SS and HA in FXS is critical for comprehensive identification of needs and appropriate interventions.

Clinical manifestations of SS have been recognized in a variety of populations for over 60 years. SS are mainly evident by behavioral responses and triggered by a variety of stimuli and environmental situations (Ayres, 1972; Cascio, 2010; McCormick et al., 2016; Zimmer et al., 2012). Although terminology and definitions vary, SS encompass abnormalities in detecting, processing, responding to, and integrating sensory stimuli into meaningful information, action, and adaptation (Bundy & Lane, 2020). In 2013, some specific SS were included as diagnostic criteria for ASD in the Diagnostic and Statistical Manual of Mental Disorders—5th Edition (DSM-5) (American Psychiatric Association, 2013). SS occur in 45–95% of individuals with ASD. Up to 13.7% of incoming kindergarteners and over 80% of children with ASD are reported to have SS (Ahn et al., 2004; Tomchek & Dunn, 2007). SS are reported in populations (Smith Roley et al., 2001), such as premature babies (Crozier et al., 2016), children with ADHD (Little et al., 2018), and children with prenatal drug and alcohol exposure (Jirikowic et al., 2020). Other genetic conditions are associated with increased SS (Galiana-Simal et al., 2020; Heald et al., 2020; Lyons-Warren et al., 2022; Neklyudova et al., 2022; Smith Roley et al., 2001).

In keeping with a recent review of sensory processing and sensory integration (SI) difficulties in ASD, we will use SS in this study as an overarching term for sensory processing disorder, sensory integration (SI) problems, sensory modulation disorder, sensory hypersensitivity/hyperreactivity, or sensorimotor deficits (Ben-Sasson et al., 2019; Lane et al., 2019). SS may include sensitivity to sounds, clothing, light touch, movement, and oral inputs such as food. SS may also include perceptual and sensory-based motor planning difficulties. Tactile defensiveness and poor eye contact were reported to be important clinical features of FXS over 30 years ago (Baranek et al., 2002; Hagerman et al., 1986, 1991; Kolacz et al., 2018; Raspa et al., 2018), but now SS are recognized to involve all senses. Miller et al. (1999) identified a lack of habituation with increased sensitization in FXS compared with those with ASD. Additionally, a study of the developmental trajectory of SS in young males with FXS demonstrated SS from infancy that grew to be more problematic through the preschool age (Baranek et al., 2008). These research findings are consistent with general clinical reports across age and sex (Hagerman & Hagerman, 2002).

Auditory, tactile, and visual paradigms are the most commonly utilized approaches to document the presence and impact of SS in FXS. For example, an exaggerated startle to auditory input with reduced habituation has been identified in individuals with FXS (Frankland et al., 2004; Hessl et al., 2009; Rais et al., 2018). These intense and poorly modulated responses to auditory stimulation, which do not diminish with exposure (e.g., hypersensitivity), are the best characterized SS phenomenon in FXS (Castren et al., 2003; Rojas et al., 2001; Rotschafer & Razak, 2014).

Another important, but less understood, clinical manifestation described in FXS is HA. HA refers to heightened physiological and psychological responses with imbalanced autonomic and emotional activation patterns (Mayes, 2000). As described by Gross et al. (2015), HA, “an over-reaction to sensory input, can be triggered in FXS by a wide range of situations, including noises, new environments, crowds, interpersonal distance, eye contact and new people. The effects of HA are widespread, and include high levels of motor activity (e.g., running, jumping), stereotypic motor movements (e.g., hand-flapping), gaze aversion, and perseverative behavior.” There may be evidence of poor autonomic reactivity (e.g., poor temperature regulation and face reddening), emotional regulation difficulties, and poor self-regulation that may include self-injurious and/or aggressive behaviors (Gross et al., 2015; Heilman et al., 2011). HA is present in individuals with FXS to varying degrees and results in limitations to the individual’s ability to participate in normal daily activities and family functioning (Gross et al., 2015).

Disorders of arousal have been described in ASD since the 1960s (Hutt et al., 1964) and continue to be explored in ASD (Hyde & Garcia-Rill, 2019). Numerous researchers have studied aspects of the biological underpinnings of HA in FXS. Roberts et al. (2001) reported on a lack of coordination between sympathetic and parasympathetic activity in response to activity demand and challenge. Heilman et al. (2011) also reported atypical autonomic activity and reactivity in FXS that increased abnormally in response to sensory and social stimulation. Klusek et al. (2015) reported faster heart rates at baseline suggesting that this heightened physiological state exists irrespective of social context. Hessl et al. (2002) reported increased cortisol levels in FXS and provided evidence that the hypothalamic–pituitary–adrenal (HPA) axis may be an independent cause of behavior problems in children with FXS. Watson et al. (2008) identified specific over activation in neural regions associated with anxiety and heightened perception and arousal. Arousal difficulties associated with shared social eye gaze may be a meaningful aspect of the FXS phenotype (Bruno et al., 2014; Klusek et al., 2020). Klusek et al. (2013) reported that arousal regulation deficits are associated with pragmatic language deficits in FXS and ASD.

While both SS and HA have been described as separate hallmark features of FXS (Cohen et al., 2015; Ethridge et al., 2017; Klusek et al., 2013), of relevance to this research, Cohen’s hyperarousal hypothesis (1995) suggested that SS and HA are interrelated. Subsequently, Belser and Sudhalter (1995) demonstrated the link between the SS of aversive eye gaze and HA in response to shared social gaze, a key social function that is atypical in FXS. Further, Black et al. (2021) reported multimodal behavioral and physiological convergence of a HA profile in infants with FXS. They suggested that HA may underlie social anxiety in young children with FXS, providing partial confirmation of the hyperarousal hypothesis (Cohen, 1995). It appears that in FXS, the influx of sensory stimulation may serve to kindle heightened physiological and emotional reactivity and responsivity, resulting in overarousal or HA (Kolacz et al., 2018) and further increasing sensory over responding.

These longstanding concerns about SS and signs of HA led to the inclusion of seven questions (Qs) into the Clinician Report Form (CRF) of the Fragile X Online Registry with Accessible Research Database (FORWARD), to learn more about them in relation to FXS. The goals of this project were to identify the prevalence of representative aspects of SS and signs of HA in FXS and to understand if these features were associated with limited participation in everyday activities. Additionally, we were interested in knowing whether SS and HA co-occur with each other or other behavior problems. Finally, we wanted to learn whether and how these conditions were being treated.

## Methods

Data analyzed for this report were derived from the FORWARD project (Sherman et al., 2017), which collected baseline and longitudinal data from 2012 to 2017 from 1070 individuals with FXS participating at 25 Fragile X Clinical and Research Consortium (FXCRC) Clinics across the United States. These analyses were conducted on the cross-sectional baseline dataset from FORWARD Version 3.0. Data were analyzed on all 933 individuals with values for the key outcome variables and basic demographics. The study was approved by the Institutional Review Board (IRB) for each participating FXCRC Clinic, and written informed consent was obtained from primary caregivers or adult patients who were their own guardians.

The Registry Form collected demographic data including age, sex, race and ethnicity, while the longitudinal database included a Parent Report Form (PRF), which was filled out by a parent or guardian; a Clinician Report Form (CRF), which was completed by a clinician with the assistance of the parent, subject, or guardian (forms described in Sherman et al., 2017); and three standardized behavioral assessments. One of them, the Aberrant Behavior Checklist-Community (ABC-C) (Aman et al., 1985) was used for these analyses. The PRF, CRF, and the ABC-C were completed annually if possible. The longitudinal database was limited to subjects with the *FMR1* full mutation with and without mosaicism. Because this information was collected at the time of a clinic visit, all answers to the questions on the CRF in FORWARD were supported by clinical observation and available supporting documentation.

The data for this report were largely derived from 10 questions (Qs) that are listed in Table 1. Three involved SS (Q66, Q68, Q69) and one involved HA (Q67). Another question from the behavior section (Q47c) was similar to (Q67) and asked whether the patient had these behaviors: hypersensitivity/overreactivity to stimuli/emotionally reactive. We included (Q47c) to provide more information on signs of HA. Two questions asked if behaviors were a limiting problem or restricted participation in daily activities (Q48c, Q72). Three asked about treatment including psychotropic medications or investigational drugs (Q49c), treatment for sensory problems (Q70), and specific interventions such as SI therapy (SIT) (Q71). For the multivariate regression models, we chose the most directly observable and least theoretical question on SS (Q66) and signs of HA (Q67) to try to learn how SS and HA related to other variables.

**Table 1:** Questions (Qs) evaluated from the Clinician Report Form

Q47: Does the child currently have this behavior?
a. Attention problems (Yes/No)
b. Hyperactivity (Yes/No)
c. Hypersensitivity/overreaction to stimuli/emotionally reactive (Yes/No)
d. Anxiety (Yes/No)
e. OCD/perseverative behavior (Yes/No)
f. Mood swings/depression (Yes/No)
g. Irritability/aggression/agitation/self-injury (Yes/No)
Q48c: Is this behavior (Q47c) a limiting problem for the child/family (e.g., going to grocery stores, birthday parties, into the community, eating in a restaurant, etc.)? (Yes/No)
Q49: Is the child on any psychopharmacological medications or investigational drugs for behaviors? (Yes/No)
Q66: Does the child respond too strongly to sensory information in his/her environment (upset by fire alarms, upset by light touch, upset by certain clothing textures, upset by certain foods)? (check one) (Never, Sometimes, Often, Always)
Q67: Does the child show signs of hyperarousal (is easily overloaded, is easily overwhelmed, is unable to cope, is unable to regulate emotions, is easily upset, has frequent outbursts, gets aggressive, becomes withdrawn, becomes socially anxious, becomes perseverative, or becomes avoidant)? (check one) (Never, Sometimes, Often, Always)
Q68: What sensory problem(s) does the child have? (check all that apply)
Tactile defensiveness
Sensitivity to certain sounds
Gravitational insecurity
Difficulty with bright lights
Difficulty with eye gaze
Other (please specify)
None
Q69: Does the child have unusual sensory input or sensory seeking behaviors (rocks, flaps hands, bites hands, jumps, bounces, walks on toes, overstuffs mouth)? (check one) (Never, Sometimes, Often, Always)
Q70: Is the child being treated for sensory problems? (check one) (Yes/No/Don’t know)
Q71: If Yes (to Q70), what specific intervention(s) are being used? (check all that apply)
Occupational/Physical Therapy
Sensory Integration Therapy
Sensory Diet
Therapeutic Listening/Auditory Training
Behavioral or Cognitive Behavioral Therapy
Medications
Other (please specify)
Q72: Does the child’s sensory problems and hyperarousal restrict participation in everyday activities in the family (e.g., going to grocery stores, going to birthday parties, going into the community, being in an inclusive setting, eating in a restaurant, spending time with friends and family)? (check one) (Never, Sometimes, Often, Always)

To evaluate factors potentially associated with SS and signs of HA, numerous variables were also included in the analyses (Table 2). The level of intellectual function was based on IQ scores and adaptive scores, information about classroom placement, and clinical judgment. There were seven possible choices for the level of intellectual functioning with the option of not answering if the clinician did not have adequate information. It was also possible to answer developmental delay (DD) if the child was under 6 years and data to substantiate level of intellectual function were not available. The presence of ASD was supported by information from patient records and the clinician’s clinical judgment. Information was gathered on the presence of behavioral problems in seven areas: attention problems; hyperactivity; hypersensitivity/overreactivity/emotionally reactive; anxiety; OCD/perseverative behavior; mood swings/depression; and irritability/aggression/agitation/self-injury (Q47a–g). An important behavioral outcome variable was the ABC-C Irritability (ABC-C-I) subscale score.

To understand comorbidities associated with a strong sensory response Q66 (Table 4), the decision was made to combine three possible answers Sometimes, Often, and Always as a positive response (“any strong sensory response”), and to use the Never answer as a negative response (“no strong sensory response”). We also examined associations between various predictor variables and “any” versus “no” strong sensory response by sex. The Sometimes response was included in the endorsement of SS but the results reported in Table 3 allow the reader to review responses in different groupings. Sometimes was accepted as a positive endorsement to capture intermittent negative outbursts or reactions.

Frequency tabulations and proportions, for categorical variables, and means and standard deviations, for continuous variables, were used for the descriptive analyses. The Chi-square test for association was used to assess differences in proportions, the Wilcoxon rank-sum test or the Kruskal–Wallis test was used for differences in ordinal variables, and the Student t-test was used for differences between mean values. For the above analyses, which are reported in Tables 3 and 4, the Bonferroni correction was used to set the alpha level for statistical significance due to multiple comparisons; p-values less than 0.002 were considered statistically significant.

To understand predictors of poor outcomes for individuals who responded too strongly to sensory information, linear regression models were fit with the ABC-C-I, reflecting aberrant behavior and irritability as the outcome, and logistic regression models were fit with the log-odds of restricted participation in everyday activities (reflecting poor quality of life) as the outcome, each with SS (Q66) as the independent variable. For these regression models, the aforementioned dichotomization of the SS predictor was used (“any” vs. “no” strong sensory response).

In addition, to study the individuals who appeared to be the most definitely impacted by SS and signs of HA, we created a new variable for each symptomatology (SS and signs of HA), that allowed a composite using additional data sources. This new composite variable was designed to add information from other questions on symptomatology to develop a more restrictive predictor of outcomes (the “Conservative Composite”). For the SS “Conservative Composite” variable, we included Q66 (Often, Always responses) with Q68 (any two sensory problems from the list presented excluding Other and None); the null or comparison group was a Never or Sometimes response to Q66 and None, Other or only one of the positive sensory responses to Q68. For the HA “Conservative Composite” variable, we included Q67 (Often, Always) with Q47c (a Yes response); the null or comparison group was a Never or Sometimes response to Q67 and a No response to Q47c.

Adjusted models included several covariates, chosen based on a priori content knowledge about the associations between the chosen covariates and outcomes of interest (Kaufmann et al., 2017; Sherman et al., 2017). These covariates were as follows: sex, age at initial study visit, level of ID, presence of hyperactivity, ASD status, and presence of anxiety. For the ID variable, subjects were grouped into four ID levels—no ID/borderline ID (reference group), mild ID, moderate ID, and severe/profound ID—and the variable was treated as a categorical predictor. Individuals, who were not classified by IQ level or reported only as having DD, were not included in the multivariable regression models. Odds ratios and 95% confidence intervals were calculated from unadjusted and adjusted models. Analyses were performed using SAS version 9.4 (SAS Institute Inc., Cary, NC, USA).

## Results

Table 2 depicts the demographic and clinical features of the sample of individuals from FXS clinics in the U.S. (Sherman et al., 2017). It was predominantly non-Hispanic white (79%), with a majority of caregivers having at least a bachelor’s degree (65%), and a relatively high family income (almost 50% with income at least $75,000). Of relevance to the characterization of SS and HA in FXS, clinicians endorsed the following related behavior problems: anxiety (81%), attention problems (79%), hypersensitivity/overreaction to stimuli/emotionally reactive (69%), hyperactivity (57%), OCD/perseverative behavior (55%), and irritability/aggression/agitation/self-injury (50%). Mood swings/depression were not highly endorsed (18%). Overall, almost 97% of the 897 individuals with complete data on each behavior presented with at least one of the above behavioral problems. Sixty-five percent of individuals were on psychopharmacological medication or investigational drugs, with males using more medication than females (67% vs. 56%, p = 0.0026). Medication use increased with age (p < 0.0001) for both males and females (Table 3). Forty-seven percent of males and 19% of females had a clinical diagnosis of ASD (Table 2).

**Table 2:** Demographic and clinical characteristics of FORWARD sample

Variables	Total sample (n = 933)		Males (n = 720)		Females (n = 213)	
	n	%	n	%	n	%
Ethnicity/race						
Hispanic/Latino	103	11.0	74	10.3	29	13.6
Non-Hispanic White	741	79.4	578	80.3	163	76.5
Non-Hispanic Black/African-American	64	6.9	50	6.9	14	6.6
Asian	17	1.8	12	1.7	5	2.3
Other	8	0.9	6	0.8	2	0.9
Age at clinical evaluation (standard deviation)	933	12.2 (8.5)	720	12.4 (8.6)	213	11.9 (7.9)
Highest level of education completed by primary guardian						
Less than high school	9	1.3	4	0.8	5	3.4
Some high school	3	0.4	3	0.6	0	0.0
High school degree or equivalent (GED)	67	10.0	53	10.2	14	9.4
Technical school/some college/associate of arts degree	156	23.3	126	24.2	30	20.1
College degree (bachelor's degree)	233	34.8	176	33.8	57	38.3
Post-graduate degree (master’s/doctorate)	200	29.9	157	30.2	43	28.9
Do not know	1	0.1	1	0.2	0	0.0
Annual household income of the primary guardian						
< $25,000	63	9.4	47	9.0	16	10.7
$25,000 to $49,999	107	15.9	81	15.5	26	17.4
$50,0000 to $74,999	121	18.0	98	18.7	23	15.4
$75,000 to $99,9999	87	12.9	65	12.4	22	14.8
$100,0000 to $149,9999	111	16.5	86	16.4	25	16.8
$150,0000 or more	124	18.5	96	18.4	28	18.8
I choose not to answer this question	59	8.8	50	9.6	9	6.0
Q47 Does the child currently have this behavior? (Yes or No)						
a. Attention problems (Yes)	924	78.6	714	80.0	210	74.3
b. Hyperactivity (Yes)	922	57.4	713	63.7	209	35.9
c. Hypersensitivity/overreaction to stimuli/emotionally reactive (Yes)	916	68.8	707	73.3	209	53.6
d. Anxiety (Yes)	919	81.4	709	80.5	210	84.3
e. OCD/perseverative behavior (Yes)	913	55.2	705	58.9	208	42.8
f. Mood swings/depression (Yes)	914	17.7	708	14.1	206	30.1
g. Irritability/aggression/agitation/self-injury (Yes)	916	49.6	707	54.6	209	32.5
Any of the behaviors listed in Q47 (Yes or No)						
Yes	897	96.6	694	96.8	203	95.8
Is the child on any psychopharmacological medications or investigational drugs for behaviors? (Yes or No)						
Yes	576	64.6	463	67.2	113	55.7
Which of these terms best describes the intellectual function?						
No intellectual disability (ID)	55	6.2	6	0.9	49	24.4
Developmental delay	109	12.3	90	13.2	19	9.5
Borderline ID	81	9.2	26	3.8	55	27.4
Mild ID	199	22.5	150	22.0	49	24.4
Moderate ID	376	42.6	351	51.5	25	12.4
Severe ID	61	6.9	57	8.4	4	2.0
Profound ID	2	0.2	2	0.3	0	0.0
Clinical diagnosis of ASD (Yes or No)						
Yes	352	40.7	314	47.4	38	18.9

Four questions related to SS and signs of HA were rated as Never, Sometimes, Often or Always. These questions remained as is for descriptive comparisons by sex and age groups (Table 3). The age groups were determined a priori to reflect infancy and toddlerhood (0–3), preschool-age (4–6), middle childhood (7–12), and teen years and beyond (13+).

**Table 3:** Responses to sensory symptoms (SS) and signs of hyperarousal (HA); Qs by gender and by age

Variables	Males											Females											Gender comparison p-value^d^
	0–3 (n = 92)		4–6 (n = 136)		7–12 (n = 215)		13 + (n = 277)		Age comparison	Total (n = 720)		0–3 (n = 17)		4–6 (n = 38)		7–12 (n = 85)		13 + (n = 73)		Age comparison	Total (n = 213)		
	n	%	n	%	n	%	n	%	p-value^d^	n	%	n	%	n	%	n	%	n	%	p-value^d^	N	%	
Q66: Does the child respond too strongly to sensory information?									0.0063^a^											0.3421^a^			< 0.0001*^b^
Never	26	28.3	13	9.6	18	8.4	36	13.0		93	12.9	7	41.2	11	28.9	22	25.9	28	38.4		68	31.9	
Sometimes	38	41.3	67	49.3	109	50.7	153	55.2		367	51.0	6	35.3	18	47.4	43	50.6	33	45.2		100	46.9	
Often	20	21.7	43	31.6	74	34.4	62	22.4		199	27.6	4	23.5	6	15.8	17	20.0	9	12.3		36	16.9	
Always	8	8.7	13	9.6	14	6.5	26	9.4		61	8.5	0	0.0	3	7.9	3	3.5	3	4.1		9	4.2	
Q67: Does the child show signs of HA?									< 0.0001*^a^											0.0100^a^			< 0.0001*^b^
Never	18	19.6	6	4.4	13	6.0	18	6.5		55	7.7	8	47.1	3	7.9	15	17.6	18	24.7		44	20.7	
Sometimes	45	48.9	65	48.1	85	39.5	170	61.6		365	50.8	7	41.2	22	57.9	43	50.6	37	50.7		109	51.2	
Often	20	21.7	50	37.0	98	45.6	69	25.0		237	33.0	2	11.8	8	21.1	20	23.5	15	20.5		45	21.1	
Always	9	9.8	14	10.4	19	8.8	19	6.9		61	8.5	0	0.0	5	13.2	7	8.2	3	4.1		15	7.0	
Q69: Does the child have unusual sensory input or sensory seeking problems?									< 0.0001*^a^											< 0.0001*^a^			< 0.0001*^b^
Never	8	8.7	6	4.4	20	9.4	42	15.3		76	10.6	8	47.1	9	23.7	25	29.4	50	69.4		92	43.4	
Sometimes	30	32.6	55	40.7	85	39.9	144	52.4		314	43.9	4	23.5	16	42.1	44	51.8	16	22.2		80	37.7	
Often	43	46.7	47	34.8	87	40.8	70	25.5		247	34.5	5	29.4	12	31.6	14	16.5	4	5.6		35	16.5	
Always	11	12.0	27	20.0	21	9.9	19	6.9		78	10.9	0	0.0	1	2.6	2	2.4	2	2.8		5	2.4	
Q72: Do sensory problems and HA restrict participation in everyday activities?									< 0.0001*^a^											0.0852^a^			< 0.0001*^b^
Never	37	40.7	25	18.4	40	18.9	83	30.3		185	25.9	11	64.7	15	39.5	38	44.7	42	57.5		106	49.8	
Sometimes	31	34.1	66	48.5	97	45.8	146	53.3		340	47.7	5	29.4	16	42.1	40	47.1	26	35.6		87	40.8	
Often	16	17.6	27	19.9	57	26.9	32	11.7		132	18.5	1	5.9	6	15.8	6	7.1	5	6.8		18	8.5	
Always	7	7.7	18	13.2	18	8.5	13	4.7		56	7.9	0	0.0	1	2.6	1	1.2	0	0.0		2	0.9	
Q47c: Does the child currently have hypersensitivity/overreactivity?									0.0010*^c^											0.0615^c^			< 0.0001*^d^
Yes	55	59.8	110	81.5	160	77.7	193	70.4		518	73.3	7	43.8	23	62.2	52	61.2	30	42.3		112	53.6	
No	37	40.2	25	18.5	46	22.3	81	29.6		189	26.7	9	56.3	14	37.8	33	38.8	41	57.7		97	46.4	
Q48c: Is hypersensitivity/overreactivity a limiting problem for the child/family?									< 0.0001*^c^											0.1234 ^c^			0.0063 ^d^
Yes	31	40.3	72	60.5	104	55.0	95	38.2		302	47.6	2	20.0	15	45.5	30	42.3	18	27.3		65	36.1	
No	46	59.7	47	39.5	85	45.0	154	61.8		332	52.4	8	80.0	18	54.5	41	57.7	48	72.7		115	63.9	
Q49: Is the child on any psychopharmacological medications?									< 0.0001*^c^											< 0.0001*^c^			0.0026 ^d^
Yes	19	21.1	62	47.7	169	81.3	213	81.6		463	67.2	1	6.3	17	47.2	47	57.3	48	69.6		113	55.7	
No	71	78.9	68	52.3	39	18.8	48	18.4		226	32.8	15	93.8	19	52.8	35	42.7	21	30.4		90	44.3	

*Indicates statistical significance

^a^Kruskal–Wallis Test for association between sensory problems and age groups within gender

^b^Wilcoxon Rank-Sum Test for association between sensory problems and gender (irrespective of age group)

^c^Chi-Squared Test for association between sensory problems and age groups within gender

^d^Chi-Squared Test for association between sensory problems and gender (irrespective of age group)

^e^Bonferroni level of correction with statistical significance set at 0.002)

Table 3 displays the age and sex distribution of SS- and HA-related features. There were prevalent and persistent difficulties with SS (Q66, Q69) and signs of HA (Q67) in males and females across age groups, although prevalence was higher in males. For Q66, 87% of males vs. 68% of females were reported to have SS (p < 0.0001). There was an increase in any SS (Q66) in males after age 3 from 72% in the 0–3 years range peaking to over 90% in the 4–12 years group. In females, 59% in the 0–3 years range were reported to have SS compared to 71% in the 4–6 years group and 74% in the 7–12 years group with a decrease in frequency to 62% after age 12. Males were also more severely impacted (Q66, Often and Always: 36% males vs. 21% females). There was a modest decrease in severity by age 13 + (fewer Often or Always responses) for both males and females (32% vs. 16%).

For Q67, 92% of males and 79% of females were reported to show signs of HA (p < 0.0001). For males, signs of HA showed a similar pattern to SS, with an increase after age 3. Signs of HA peaked in females during ages 4–12. In terms of severity, signs of HA were endorsed Often and Always for 42% of the males vs. 28% of females. For Q47c (Table 3), hypersensitivity/overreaction/emotionally reactive behavior was also endorsed for 73% of males and 54% of females (p < 0.0001). As with SS and signs of HA, the behavior problems worsened after age 3, but they were reported to improve in females after age 12.

For Q69, 89% of males and 57% of females were reported to have any unusual sensory input or sensory seeking problems behaviors (p < 0.0001). While in males the frequency of these remained relatively stable across ages, in females these behaviors increased between 4–12 years and showed a sharp decrease in adolescence with only 31% of the females over age 12 reported to have these sensory behaviors. In terms of severity (Often or Always responses), Q69 was endorsed 45% for males compared to 19% for females. Two questions (Q48c and Q72, Table 3) were designed to identify whether there was a negative impact to having the problems related to SS and HA. For Q48c, hypersensitivity/overreaction to stimuli/emotionally reactive behaviors were a limiting problem for the child/family 48% of the time for males and 36% for females. For Q72, SS/HA restricted participation in everyday activities 74% of the time for males and 50% for females.

The two key questions on SS (Q66, Q69) were significantly correlated (r = 0.45, p < 0.0001). Additionally, HA (Q67) was highly associated with each of the SS questions with correlation coefficients of 0.61 and 0.49, respectively, p < 0.0001. Similarly, there was a high correlation between endorsing SS or signs of HA and whether these behaviors were limiting for the family (Q72). These correlation coefficients ranged from 0.53 to 0.61 (p < 0.0001). Q47c correlated with all four questions discussed above (p < 0.0001).

**Fig. 1:**
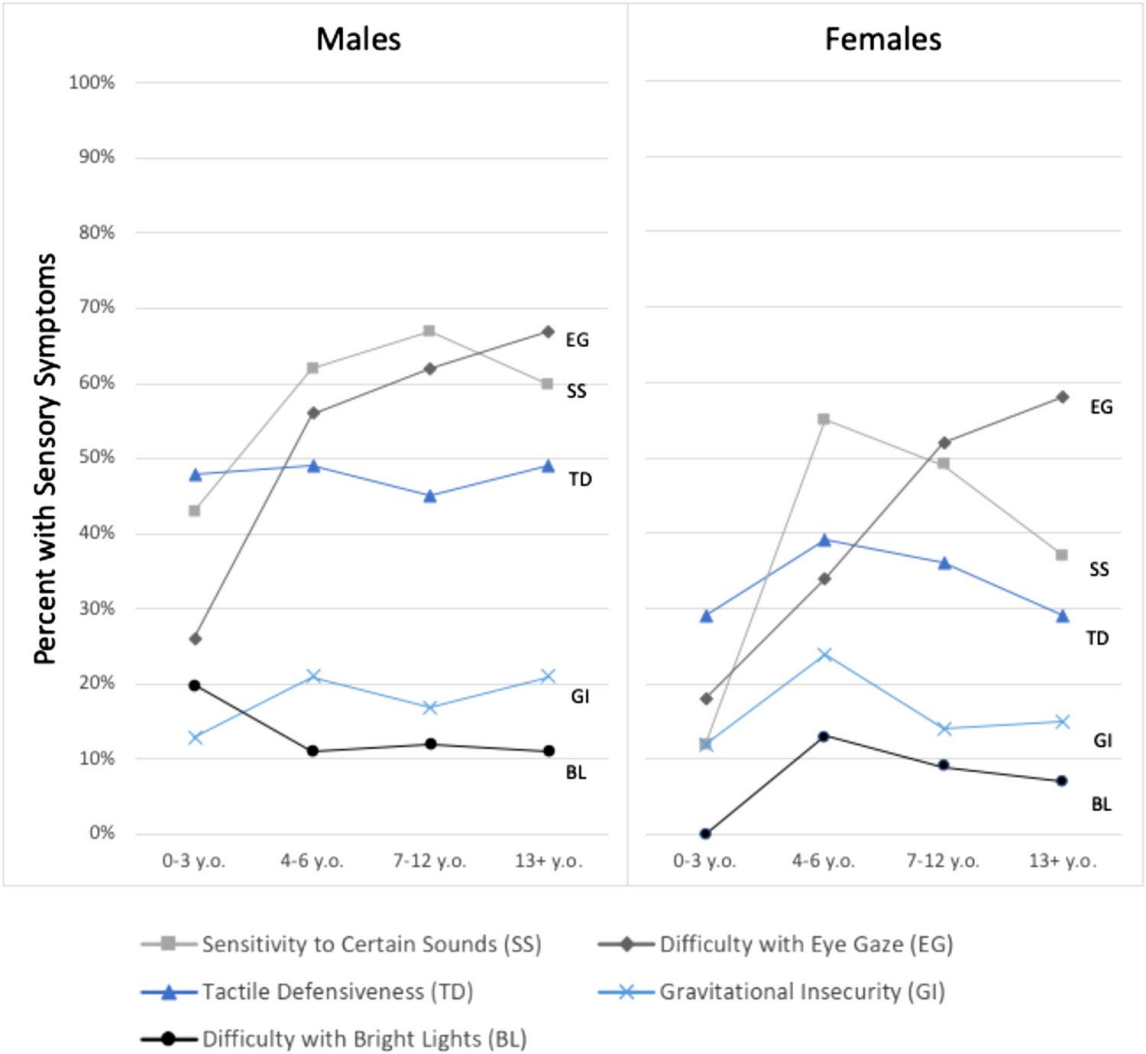
Sensory symptoms endorsed by sex and by four age groups

In terms of specific SS, as shown in Fig. 1, a high proportion of males (56%) and females (46%) had difficulty with eye gaze. This was the only problem that appeared to increase substantially with age, with 65% of males and 56% of females ages 13 and over having it. Difficulty with eye gaze was highly correlated with SS, signs of HA, anxiety, inattention and ASD (p < 0.0001; data not shown), and less so with hyperactivity (p = 0.0108). Sensitivity to certain sounds was reported in 59% of males and 42% of females), with a sharp increase in this problem after age 3 and a decrease after age 12 for the males and after age 6 for the females. Tactile defensiveness was endorsed for 46% of males and 34% of females with a prevalence fairly consistent across age ranges for males and peak at 4–6 years for females. Gravitational insecurity (17%) and difficulty with sensitivity to bright lights (11%) were reported less frequently, with no significant difference between males and females.

**Fig. 2:**
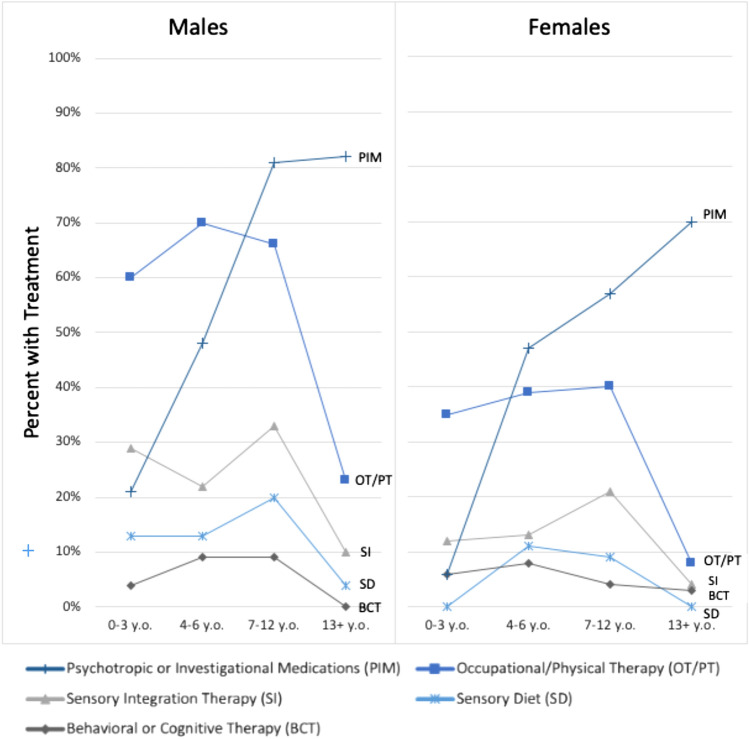
Treatments endorsed by sex and by four age groups

Forty-eight percent of the total sample were reported as being treated for sensory problems by one of the treatments or therapies shown in Fig. 2. The most prevalent treatment was OT/PT, which was being received by 44% of the entire sample. Through age 12, 64% of males and 38% of females received OT/PT services, but after age 12, OT/PT treatment was markedly reduced to 24% of males and 9% of females. Other treatments for SS, such as sensory integration therapy (SIT) and sensory diet, were used less than OT/PT and decreased after age 12. Nineteen percent of clinicians endorsed that SIT was being used and 10% endorsed that a sensory diet was being used. Males used SIT and sensory diets in greater proportion at all ages. Therapeutic listening/auditory training was only endorsed 1% of the time. In contrast, as age increased, so did the use of psychopharmacological or investigational medications (p < 0.0001). By age 7 and over, more than 81% of males were using medications and by age 13, 69.6% of females (Q49 in Table 3).

**Table 4:** Likelihood of comorbidities when a strong sensory response is endorsed

Variables	Males					Females				
	Any strong sensory response		No strong sensory response		p-value^a,b^	Any strong sensory response		No strong sensory response		p-value^a,b^
	n	%	n	%		n	%	n	%	
Age at clinical evaluation—mean ± standard deviation	627	12.5 ± 8.6	93	11.3 ± 8.9	0.1891	145	11.4 ± 7.5	68	13.0 ± 8.8	0.1693
Intellectual disability (ID)	590		92		< 0.0001*	139		62		< 0.0001*
No ID	2	0.3	4	4.3		21	15.1	28	45.2	
Developmental delay	69	11.7	21	22.8		13	9.4	6	9.7	
Borderline ID	18	3.1	8	8.7		40	28.8	15	24.2	
Mild ID	131	22.2	19	20.7		40	28.8	9	14.5	
Moderate ID	314	53.2	37	40.2		22	15.8	3	4.8	
Severe or profound ID	56	9.5	3	3.3		3	2.2	1	1.6	
Clinical diagnosis of ASD (Yes or No)	577		86		0.0003*	135		66		0.0130
Yes	289	50.1	25	29.1		32	23.7	6	9.1	
No	288	49.9	61	70.9		103	76.3	60	90.9	
ABC-C Irritability Subscale—mean ± standard deviation	473	13.0 ± 10.1	65	6.5 ± 7.5	< 0.0001*	113	10.4 ± 9.2	45	5.7 ± 7.5	0.0026
Q47a: Does the child currently have attention problems?	621		93		0.0039	143		67		0.1060
Yes	507	81.6	64	68.8		111	77.6	45	67.2	
No	114	18.4	29	31.2		32	22.4	22	32.8	
Q47b: Does the child currently have hyperactivity?	620		93		0.0331	142		67		0.0295
Yes	404	65.2	50	53.8		58	40.8	17	25.4	
No	216	34.8	43	46.2		84	59.2	50	74.6	
Q47c: Does the child currently have hypersensitivity/overreactivity/emotionally reactive?	614		93		< 0.0001*	142		67		< 0.0001*
Yes	491	80.0	27	29.0		104	73.2	8	11.9	
No	123	20.0	66	71.0		38	26.8	59	88.1	
Q47d: Does the child currently have anxiety?	617		92		< 0.0001*	144		66		0.0587
Yes	518	84.0	53	57.6		126	87.5	51	77.3	
No	99	16.0	39	42.4		18	12.5	15	22.7	
Q47e: Does the child currently have OCD/perseverative behavior?	614		91		< 0.0001*	141		67		0.0014*
Yes	380	61.9	35	38.5		71	50.4	18	26.9	
No	234	38.1	56	61.5		70	49.6	49	73.1	
Q47f: Does the child currently have mood swings/depression?	616		92		0.1999	139		67		0.0940
Yes	91	14.8	9	9.8		47	33.8	15	22.4	
No	525	85.2	83	90.2		92	66.2	52	77.6	
Q47g: Does the child currently have irritability/aggression/agitation/self-injury?	615		92		< 0.0001*	142		67		0.0019*
Yes	357	58.0	29	31.5		56	39.4	12	17.9	
No	258	42.0	63	68.5		86	60.6	55	82.1	

*Indicates statistical significance

^a^Chi-Squared Test for association between comorbidity and strong response within gender

^b^Bonferroni level of correction with statistical significance set at 0.002

Table 4 demonstrated that endorsing strong sensory responses (Q66) was correlated with several predictors in both sexes. Both males and females with any strong sensory response were more impacted by ID (both at p < 0.0001) while males had twice as high of a score on the ABC-C-I subscale and females had almost twice as high of a score (males: 13.0 ± 10.1 vs. 6.5 ± 7.5, p < 0.0001; females: 10.4 ± 9.2 vs. 5.7 ± 7.5, p = 0.0026). Males and females with any strong sensory response were significantly more likely to have a clinical diagnosis of ASD (males: 50.1% vs. 29.1%, p = 0.0003; females: 23% vs. 9.1%, p = 0.013), but this was not significant for females after Bonferroni correction.

For both sexes, endorsement of three behavioral categories including hypersensitivity/overreaction to stimuli/emotionally reactive, OCD/perseverative behavior, and irritability/aggression/agitation/self-injury were associated with having any strong sensory response (p < 0.002). Mood swings/depression was not significantly associated with having any strong sensory response for either males (p = 0.1999) or females (p = 0.094). Endorsement of hyperactivity was not associated with any strong sensory response for males (p = 0.0331) or females (p = 0.0295) after Bonferroni correction. Endorsement of attention problems was not associated with any strong sensory response using the Bonferroni correction for males (p = 0.0039) or females (p = 0.1060). Endorsement of anxiety was associated with any strong sensory response for males (p < 0.0001) but not females (p < 0.0587). Females were reported to have high levels of inattention and anxiety whether or not they had strong SS.

Multivariate regression models examining the role of strong responsivity to SS in the presence of other strong predictors of poor outcomes (Table 5) showed that analyses using the Conservative Composite variables for SS or HA were important contributors to the multivariate analyses. Using a combination of data responses rather than relying on the single SS (Q66) and HA (Q67) questions was a valuable alternative, thus providing refined hypotheses and creating more definite descriptors of these symptom complexes.

**Table 5:** Multivariate regression models—predictors of poor outcomes for individuals who responded strongly to sensory information

Model 1: Effect estimate for a high score on ABC-C Irritability Subscale						
Effect estimate for a high score on ABC-C Irritability Subscale (continuous)	1.A. Unadjusted linear regression model (Q66. respond too strongly to sensory information)		1.B. Unadjusted linear regression model (Composite Conservative variable re SS)^a^		1.C. Unadjusted linear regression model (Composite Conservative variable re signs of HA)^b^	
	Estimate (95% CI)	p-value	Estimate (95% CI)	p-value	Estimate (95% CI)	p-value
Responded too strongly to sensory information or exhibited signs of HA (See columns A, B, or C for description of main effect in each model)	6.74 (4.64, 8.84)	< 0.0001	4.93 (3.24, 6.62)	< 0.0001	7.84 (6.35, 9.34)	< 0.0001
	Adjusted linear regression model (Q66. respond too strongly to sensory information)		Adjusted linear regression model (Composite Conservative variable re SS)		Adjusted linear regression model (Composite Conservative variable re signs of HA)	
Responded too strongly to sensory information or exhibited signs of HA (See columns A, B, or C for description of main effect in each model)	3.52 (1.39, 5.65)	0.0012	2.58 (0.8, 4.36)	0.0045	5.27 (3.63, 6.91)	< 0.0001
Male vs female	0.76 (− 1.45, 2.97)	0.4978	1.00 (− 1.22, 3.21)	0.3770	0.73 (− 1.42, 2.88)	0.5047
Age at visit	− 0.21 (− 0.31, − 0.12)	< 0.0001	− 0.22 (− 0.31, − 0.12)	< 0.0001	− 0.19 (− 0.28, − 0.09)	< 0.0001
Level of intellectual disability (ID) (mild ID vs no ID or borderline ID)	0.75 (− 1.83, 3.33)	0.5703	0.99 (− 1.58, 3.56)	0.4497	0.91 (− 1.58, 3.41)	0.4727
Level of ID (moderate ID vs no ID or borderline ID)	1.36 (− 1.36, 4.09)	0.3262	1.52 (− 1.21, 4.25)	0.2748	1.73 (− 0.91, 4.37)	0.1983
Level of ID (severe or profound ID vs no ID or borderline ID)	5.75 (1.96, 9.55)	0.0030	5.65 (1.83, 9.47)	0.0038	5.78 (2.08, 9.49)	0.0023
Hyperactivity	3.49 (1.87, 5.10)	< 0.0001	3.54 (1.92, 5.16)	< 0.0001	2.75 (1.15, 4.35)	0.0008
ASD by clinician diagnosis	3.57 (1.87, 5.28)	< 0.0001	3.26 (1.52, 4.99)	0.0003	2.87 (1.19, 4.55)	0.0009
Anxiety	3.34 (1.13, 5.55)	0.0031	3.57 (1.36, 5.77)	0.0016	2.46 (0.28, 4.64)	0.0271

Model 2: Effect estimate for restricted participation in everyday activities (Q72)						
Odds ratios of restricted participation in everyday activities (Yes/No)	2.A. Unadjusted logistic regression model (Q66 respond too strongly to sensory information)		2.B. Unadjusted logistic regression model (Composite Conservative variable re SS)^a^		2.C. Unadjusted logistic regression model (Composite Conservative variable re signs of HA)^b^	
	Estimate (95% CI)	p-value	Estimate (95% CI)	p-value	Estimate (95% CI)	p-value
Responded too strongly to sensory information or exhibited signs of HA (See columns A, B, or C for description of main effect in each model)	14.60 (9.24, 23.05)	0.0001	11.15 (6.09, 20.42)	< 0.0001	10.44 (6.35, 17.16)	< 0.0001
	Adjusted logistic regression model (Q66 respond too strongly to sensory information)		Adjusted logistic regression model (Composite Conservative variable re SS)		Adjusted logistic regression model (Composite Conservative variable re signs of HA)	
Responded too strongly to sensory information or exhibited hyperarousal (See columns A, B, or C for description of main effect in each model)	11.22 (6.60, 19.08)	< 0.0001	8.9 (4.13, 19.21)	< 0.0001	6.85 (3.82, 12.28)	< 0.0001
Male vs female	1.18 (0.068, 2.04)	0.5573	1.38 (0.84, 2.29)	0.2072	1.3 (0.77, 2.17)	0.3236
Age at visit	0.97 (0.95, 0.99)	0.0085	0.97 (0.95, 1)	0.0263	0.98 (0.96, 1)	0.0699
Level of intellectual disability (ID) (mild ID vs no ID or borderline ID)	1.84 (1.00, 3.39)	0.0519	2.29 (1.3, 4.04)	0.0044	2.58 (1.46, 4.54)	0.0011
Level of ID (moderate ID vs no ID or borderline ID)	3.35 (1.73, 6.50)	0.0003	3.77 (2.07, 6.89)	< 0.0001	4.45 (2.39, 8.29)	< 0.0001
Level of ID (severe or profound ID vs no ID or borderline ID)	4.35 (1.47, 12.86)	0.0079	4.53 (1.68, 12.23)	0.0029	5.6 (2.11, 14.88)	0.0006
Hyperactivity	1.21 (0.78, 1.87)	0.3924	1.21 (0.81, 1.82)	0.3467	1 (0.67, 1.51)	0.9822
ASD by clinician diagnosis	1.93 (1.17, 3.16)	0.0095	1.61 (1.01, 2.55)	0.0455	1.64 (1.02, 2.63)	0.0411
Anxiety	2.43 (1.40, 4.21)	0.0016	2.36 (1.46, 3.81)	0.0005	2.09 (1.3, 3.38)	0.0025

^a^Composite conservative variable for SS equals endorsing Often and Always (Q66) and two or more SS (Q68)

^b^Composite conservative variable for signs of HA equals endorsing Often and Always (Q67) and endorsing Yes for (Q47c)

The unadjusted estimate in the linear regression model for SS suggested a statistically significant and clinically important increase in the score of the ABC-C-I subscale (Model 1.A.) for those who had any strong response to sensory information. This predictor of irritability was tempered in the adjusted model by the inclusion of sex, ASD status, level of ID, presence of hyperactivity, anxiety, and age. However, the estimate remained highly significant (almost half of the unadjusted model) and clinically important (a 3.52 point score increase with respect to the unadjusted model) for those who had any strong response to sensory information. After SS, level of ID had the largest effect estimate of an increase in ABC-C-I score, although this was only observed for the most severe level of ID and the confidence interval was wider. Age at visit suggested a 0.2 decrement in ABC-C-I score (less irritability) for every increase in year of age. Having hyperactivity, ASD, or anxiety were also independent predictors of higher scores, with effect estimates similar to that of having SS (approximately 3.5) and with high significance (all at p < 0.003).

Using the Composite Conservative variable for SS (Model 1.B.), the adjusted model results observed were a combination of tempered and increased effect estimates, but still remaining with clinically important effect estimates and high levels of statistical significance. Importantly, the main effect (the Composite Conservative variable) remained strongly predictive of a higher score on the ABC-C-I subscale. Using the Composite Conservative variable for signs of HA (Model 1.C.), the main effect was predictive of a poor ABC-C-I subscale score, suggesting an increase of 5.27 points, with a confidence interval of 3.63 to 6.91 points in the adjusted model. 

In Model 2.A. (Table 5), the odds of restricted participation (Q72) was strongest for SS, although confidence intervals were wide in comparison to anxiety, ASD, age, and level of ID. The odds ratio suggested an 11-fold (11 times) odds of restricted participation in everyday activities for any strong response to sensory information, with the confidence interval suggesting at least a sixfold odds of restricted participation, adjusting for the effects of other predictors. Age at visit suggested a modest decrease in the odds of restricted participation at older ages. In this model, ID was a stronger independent predictor at all levels of ID, with a dose response indicating that the odds of restricted participation increase as the level of ID becomes more severe. Having a diagnosis of ASD or anxiety in this model also increased the odds of restricted participation by at least twofold and at a statistically significant level.

Using the Composite Conservative variable for SS (Model 2.B.), the adjusted model results observed were similar to Model 2.A., but the main effect in the unadjusted and adjusted analyses was of a lower magnitude than the SS variable in Model 2.A. ID became even more important as an independent predictor, suggesting a continued outsized role compared to the other covariates. In Model 2.C., examining the Composite Conservative variable for signs of HA, the odds of restricted participation remained very strong in both unadjusted and adjusted models, with ID continuing to be important and with a dose–response association with restricted activities—the higher the level of ID combined with the impact of SS and HA, the greater the odds of restricted participation.

## Discussion

The FORWARD project provides data on the largest clinical sample of subjects with FXS evaluated to date. For this investigation, we studied 10 questions answered by clinicians related to SS and signs of HA because these symptoms impair functioning of individuals with FXS (Bailey et al., 2008; Dominick et al., 2021; Hagerman & Hagerman, 2002; Tranfaglia, 2011; Tsiouris & Brown, 2004). SS and HA may be at the base of other frequently occurring problems such as aggression and self-injurious behaviors (Eckert et al., 2019; Hall et al., 2012; Wheeler et al., 2016). Our aims were to study SS and signs of HA prevalence, impact, and treatment across ages and sexes, and whether these problems restricted participation in everyday activities in the family. We also intended to assess SS and signs of HA in relation to neurobehavioral comorbidities such as low IQ, ASD, and behavioral concerns, as available data are limited.

SS and signs of HA were highly endorsed by clinicians far more frequently in males, which is consistent with males with FXS being more affected. SS and signs of HA increased somewhat after age 3 years, with a peak between 4 and 12 years, and persisted thereafter. This study also supports previous reports that certain SS are very prevalent in FXS, including difficulty with eye gaze, sensitivity to certain sounds, and tactile defensiveness (Q68 and Fig. 1). SS and HA limited participation in daily living activities (Q72). Endorsing SS (Q66) was associated with lower IQs for both sexes and much higher scores on the ABC-C-I subscale. Q66 was endorsed for more individuals with FXS and ASD than individuals with FXS without ASD; however, SS and signs of HA were reported in both groups.

Multiple regression models suggested that endorsing SS and signs of HA (Q66 and Q67) was associated with increased ABC-C-I subscale scores and limited participation in everyday activities such as going into the community (Table 5). Problems with SS and HA persisted particularly for males into the oldest age range (Table 3). A large percentage (44%) of individuals in this study were currently receiving OT/PT services to treat sensory problems, with a higher proportion in individuals younger than 13 (Fig. 2). The majority of subjects were not receiving SIT or using a sensory diet.

Individuals with FXS present with a variety of behavioral concerns such as inattention, hyperactivity, anxiety and aggression (Bailey et al., 2008; Dominick et al., 2021; Eckert et al., 2019; Tranfaglia, 2011; Wheeler et al., 2016). In this study, all areas of behavioral concern assessed correlated with the presence of SS and signs of HA except for mood swings/depression (Q47f). In addition to the findings presented here, that demonstrated a correlation between SS and signs of HA, there is concern that SS and HA influence each other and this may escalate the negative impacts of SS and HA exponentially (Belser & Sudhalter, 1995; Black et al., 2021; Cohen, 1995; Tsiouris & Brown, 2004). For example, difficulty with loud noises can trigger a state of HA resulting in fear, flight, or fight behaviors that are expressions of HA but potentially mislabeled as behavior problems such as anxiety.

Managing SS requires practical solutions and adaptations to avoid distressing reactions. For example, simple modifications to cope with loud noises can be helpful such as wearing noise canceling headphones or eating in a quiet setting instead of a noisy cafeteria. Often, individuals have several SS along with HA and other behavioral problems, and they will require professional assistance for optimal functioning. This is typically provided by OTs with specialized training to manage these problems. OTs aim to improve the individual’s ability to function in difficult situations rather than avoid them. Numerous treatment recommendations, including using SIT and sensory diets (Stackhouse et al., 2014; Wilbarger & Wilbarger, 1991), are available through the website of the National Fragile X Foundation (https://fragileX.org/our-research/treatment-recommendations/). In this study, the scope of OT/PT services being received was not specified and may have represented therapies provided by early intervention programs or the public school system but not necessarily tailored to individuals with FXS. SIT, specifically the Ayres Sensory Integration® intervention (ASI), is considered an evidence-based practice for children with ASD (Case-Smith et al., 2015; Schaaf et al., 2014, 2018; Shoen et al., 2019a, 2019b). Further research on the benefits of SIT is ongoing.

While, in the present study, therapeutic non-medication services decreased after age 13, there was a simultaneous increase in psychotropic medication use. We could not address the causes for this decrease in non-medication services in the older age group. Possible reasons could be lack of available services in the school system, high cost of private services, and/or perceived lack of effectiveness. This lack of services for older individuals could have health policy implications, namely the need to provide these ancillary services if they are warranted. Some medications indicated for ADHD, specifically alpha-adrenergic agonists (clonidine and guanfacine), are also useful to treat HA (Berry-Kravis & Potanos, 2004; Berry-Kravis et al.,^ 2012^; Erickson, 2021;  Hagerman et al., 1995; Hersh et al., 2011; Tsiouris & Brown, 2004). In addition, medical management of co-existing mental health problems such as hyperactivity may make it easier to treat HA and SS. Successful medication management of individuals with FXS can be difficult (Bailey et al., 2012), and a multidisciplinary approach that includes professionals skilled at managing relevant problems associated with FXS as well as SS and HA is optimal (Hagerman et al., 2009; Stackhouse et al., 2014). It is also customary that individuals with significant developmental disabilities have access to one-on-one respite care or developmental service providers. These services could increase participation in activities at home and in the community. Some individuals with both FXS and ASD may benefit from Applied Behavior Analysis (ABA), which is widely available for individuals with ASD. ABA providers generally do not have specific training to address SS and HA, however. As SS and HA are associated with mental health problems, behavioral therapy may also be helpful, but it was rarely endorsed in this set of questions (Fig. 2). In summary, this study suggests that individuals with FXS, who also have SS and signs of HA, are undertreated.

SS have been increasingly appreciated over the past 30 years (Ben-Sasson et al., 2019), and, to date, much of the work on HA in FXS is focused on physiological differences such as those involving the autonomic nervous system rather than the clinical presentation. For example, abnormal heart activity and cortisol levels have been investigated (Hessl et al., 2002; Hogan et al., 2021). Despite this increased knowledge about SS and HA in FXS, recognition and treatment of these problems may be limited. Understanding how SS and HA impact daily functioning in individuals with FXS could positively influence how we treat many behaviors ranging from anxiety to intermittent explosive disorder and aggression, especially when these behaviors are worsened by SS and/or HA. This improvement could enhance the overall quality of life for individuals with FXS. Furthermore, the insights gained from understanding these problems in FXS may also be applicable to other neurodevelopmental disorders with associated behavioral challenges.

The major shortcoming of this study was the limited scope of the questions on SS and signs of HA and their lack of validation prior to their use. The question on interventions combined OT and PT as one possible intervention, and there were no response options that described specific types of treatment. The question inquiring about the impact of SS and HA (Q72) combined these problems as well; separating them might have provided more information about the relative impact of each. We did not include a specific query regarding whether the subjects had received ABA. Since ASD co-occurs in almost 50% of males with FXS, it would be important to know if this treatment is being used and whether it is beneficial. The FORWARD project did not inquire about the use of respite care or developmental care either. Participants in FORWARD most likely represented moderately impaired individuals with FXS, because caregivers of patients with milder symptoms may not seek specialty care, and patients with severe behavioral co-occurring conditions may be too difficult to bring to a FXS clinic. Based on the demographic information, Caucasian families were overrepresented and mean educational level and SES of caregivers were higher than that of the general American population (Kaufmann et al., 2017; Sherman et al., 2017). To address these issues more appropriately, targeted recruitment of underrepresented minorities and inclusion of individuals over age 25 was eventually implemented into the FORWARD project. Forms were translated into Spanish to facilitate data acquisition from the Spanish-speaking population. Therefore, future studies may overcome the shortcomings of the data analyzed here. This study did not address the fact that, due to the rarity of this condition, multiple family members were allowed to participate in this study (15–20% percent of families depending on the inclusion/exclusion criteria). This could result in non-independence of observations and may have impacted the findings. Despite these limitations, the questions studied in this report were answered by expert clinicians, who had the advantage of evaluating the subjects personally and interviewing their caregivers. This careful questioning should supply additional evidence to support the presence of SS and signs of HA in the varying degrees that were described.

This study suggests that there is still great need for continued research on SS and HA in FXS. All aspects of SS in relation to FXS should be studied rather than only a representative sample of questions. Available standardized diagnostic measures should also be used (Kolacz et al., 2018). There is a need for more understanding about how HA manifests itself clinically and how it limits individuals; developing clinical tools for assessment of HA would be beneficial. Researchers should evaluate the efficacy of standardized OT treatments for SS and HA, as recommended by the American Occupational Therapy Association (Stackhouse et al., 2014). To improve behavior management, behavioral problems should be described considering SS and HA knowledge, identifying triggers or antecedents. Understanding the underutilization of psychotherapy for families would be valuable. The effectiveness of medications like clonidine for HA should also be studied.

Exploring the independent roles of SS and HA, as well as their relationship, is crucial, as HA may arise due to factors other than SS. Importantly, the relationship between SS and HA with other variables should continue to be studied. For example, fewer than half of males with FXS have a diagnosis of ASD, but over 80% of individuals in this study have some evidence of SS and signs of HA. This could suggest that SS and HA do not necessarily lead to a diagnosis of ASD or that ASD is underdiagnosed. There should be more research on how SS and signs of HA present in children from 0 to 3 years, since these problems occur somewhat less often in this age range. Knowledge about young children may be limited since they frequently are not diagnosed until about age 3 (Raspa et al., 2023). Continuation of research into the biological underpinnings of SS and HA is recommended, focusing on stress response systems (Contractor et al., 2015; Fung & Reiss, 2016; Hessl et al., 2002). Additionally, incorporating biomarkers to measure behavior and its changes could enhance the FXS clinical field (Berry-Kravis et al., 2013; Zafarullah & Tassone, 2019), as behavior checklists might not be as sensitive in capturing treatment initiation improvements.

In conclusion, our analysis of FORWARD project data has provided an initial but comprehensive overview of the highly prevalent SS and signs of HA in FXS as endorsed by clinicians. More research is warranted to understand the full impact of these problems, as specialized treatments appear to be indicated. Clinically relevant questions included in this study broadened our understanding of the profile of SS and HA for individuals with FXS. These data support SS and HA as core neurodevelopmental phenotypes of FXS, but more research is needed to fully understand their characteristics, associations, treatment, and impact on individuals with the disorder.

## References

[CR1] Ahn, R. R., Miller, L. J., Milberger, S., & McIntosh, D. N. (2004). Prevalence of parents’ perceptions of sensory processing disorders among kindergarten children. *American Journal of Occupational Therapy,**58*(3), 287–293. 10.5014/ajot.58.3.287 10.5014/ajot.58.3.287 15202626

[CR2] Aman, M. G., Singh, N. N., Stewart, A. W., & Field, C. J. (1985). The aberrant behavior checklist: A behavior rating scale for the assessment of treatment effects. *American Journal of Mental Deficiency,**89*(5), 485–491. 3993694

[CR3] American Psychiatric Association, DSM-5 Task Force. (2013). *Diagnostic and statistical manual of mental disorders: DSM-5™* (5th ed.). American Psychiatric Publishing. 10.1176/appi.books.9780890425596

[CR4] Ayres, A. J. (1972). *Sensory integration and learning disorders*. Western Psychological Services.

[CR5] Bailey, D. B., Jr., Raspa, M., Bishop, E., Olmsted, M., Mallya, U., & Berry-Kravis, E. (2012). Medication utilization for targeted symptoms in children and adults with fragile X syndrome: US survey. *Journal of Developmental & Behavioral Pediatrics,**33*(1), 62–69. 10.1097/DBP.0b013e318236c0e1 22064563 10.1097/DBP.0b013e318236c0e1

[CR6] Bailey, D. B., Jr., Raspa, M., Olmsted, M., & Holiday, D. B. (2008). Co-occurring conditions associated with *FMR1* gene variations: Findings from a national parent survey. *American Journal of Medical Genetics Part A,**146A*(16), 2060–2069. 10.1002/ajmg.a.32439 18570292 10.1002/ajmg.a.32439

[CR7] Baranek, G. T., Chin, Y. H., Greiss Hess, L. M., Yankee, J. G., Hatton, D. D., & Hooper, S. R. (2002). Sensory processing correlates of occupational performance in children with fragile X syndrome: Preliminary findings. *American Journal of Occupational Therapy,**56*(5), 538–546. 10.5014/ajot.56.5.538 10.5014/ajot.56.5.538 12269508

[CR8] Baranek, G. T., Roberts, J. E., David, F. J., Sideris, J., Mirrett, P. L., Hatton, D. D., & Bailey, D. B. (2008). Developmental trajectories and correlates of sensory processing in young boys with fragile X syndrome. *Physical and Occupational Therapy in Pediatrics,**28*(1), 79–98. 10.1300/J006v28n01_06 18399048 10.1300/j006v28n01_06

[CR9] Berry-Kravis, E., Hessl, D., Abbeduto, L., Reiss, A. L., Beckel-Mitchener, A. B., Urv, T. K., Outcome Measures Working Groups. (2013). Outcome measures for clinical trials in fragile X syndrome. *Journal of Developmental and Behavioral Pediatrics,**34*(7), 508–522. 10.1097/DBP.0b013e31829d1f20 24042082 10.1097/DBP.0b013e31829d1f20 PMC3784007

[CR10] Berry-Kravis, E., & Potanos, K. (2004). Psychopharmacology in fragile X syndrome—Present and future. *Developmental Disabilities Research Reviews,**10*(1), 42–48. 10.1002/mrdd.20007 10.1002/mrdd.20007 14994287

[CR11] Berry-Kravis, E., Sumis, A., Hervey, C., & Mathur, S. (2012). Clinic-based retrospective analysis of psychopharmacology for behavior in fragile X syndrome. *International Journal of Pediatrics,**2012*, 843016. 10.1155/2012/843016 22899942 10.1155/2012/843016 PMC3413981

[CR12] Belser, R. C., & Sudhalter, V. (1995). Arousal difficulties in males with fragile X syndrome: A preliminary report. *Developmental Brain Dysfunction,**8*(4–6), 270–279.

[CR13] Ben-Sasson, A., Gal, E., Fluss, R., Katz-Zetler, N., & Cermak, S. A. (2019). Update of a meta-analysis of sensory symptoms in ASD: A new decade of research. *Journal of Autism and Developmental Disorders,**49*, 4974–4996. 10.1007/s10803-019-04180-0 31501953 10.1007/s10803-019-04180-0

[CR14] Black, C. J., Hogan, A. L., Smith, K. D., & Roberts, J. E. (2021). Early behavioral and physiological markers of social anxiety in infants with fragile X syndrome. *Journal of Neurodevelopmental Disorders,**13*, 11. 10.1186/s11689-021-09356-3 33743580 10.1186/s11689-021-09356-3 PMC7980359

[CR15] Boyle, L., & Kaufmann, W. E. (2010). The behavioral phenotype of FMR1 mutations. *American Journal of Medical Genetics Part c. Seminars in Medical Genetics,**154C*(4), 469–476. 10.1002/ajmg.c.30277 20981777 10.1002/ajmg.c.30277

[CR16] Bruno, J. L., Garrett, A. S., Quintin, E., Mazaika, P. K., & Reiss, A. L. (2014). Aberrant face and gaze habituation in fragile X syndrome. *The American Journal of Psychiatry,**171*(10), 1099–1106. 10.1176/appi.ajp.2014.13111464 24969119 10.1176/appi.ajp.2014.13111464 PMC4182125

[CR17] Bundy, A. C., & Lane, S. J. (2020). *Sensory integration theory and practice* (3rd ed.). F. A. Davis Co.

[CR18] Cascio, C. J. (2010). Somatosensory processing in neurodevelopmental disorders. *Journal of Neurodevelopmental Disorders,**2*, 62–69. 10.1007/s11689-010-9046-3 22127855 10.1007/s11689-010-9046-3 PMC3164038

[CR19] Case-Smith, J., Weaver, L. L., & Fristad, M. A. (2015). A systematic review of sensory processing interventions for children with autism spectrum disorders. *Autism,**19*(2), 133–148. 10.1177/1362361313517762 24477447 10.1177/1362361313517762

[CR20] Castren, M., Paakkonen, A., Tarkka, I. M., Ryynanen, M., & Partanen, J. (2003). Augmentation of auditory N1 in children with fragile X syndrome. *Brain Topography,**15*, 165–171. 10.1023/A:1022606200636 12705812 10.1023/a:1022606200636

[CR21] Cohen, I. L. (1995). Behavioral profiles of autistic and nonautistic fragile X males. *Developmental Brain Dysfunction,**8*(4–6), 252–269.

[CR22] Cohen, S., Masyn, K., Mastergeorge, A., & Hessl, D. (2015). Psychophysiological responses to emotional stimuli in children and adolescents with autism and fragile X syndrome. *Journal of Clinical Child and Adolescent Psychology,**44*(2), 250–263. 10.1080/15374416.2013.843462 24156344 10.1080/15374416.2013.843462 PMC3999342

[CR23] Contractor, A., Klyachko, V. A., & Portera-Cailliau, C. (2015). Altered neuronal and circuit excitability in fragile X syndrome. *Neuron,**87*(4), 699–715. 10.1016/j.neuron.2015.06.017 26291156 10.1016/j.neuron.2015.06.017 PMC4545495

[CR24] Crozier, S. C., Goodson, J. Z., Mackay, M. L., Synnes, A. R., Grunau, R. E., Miller, S. P., & Zwicker, J. G. (2016). Sensory processing patterns in children born very preterm. *American Journal of Occupational Therapy,**70*(1), 1–7. 10.5014/ajot.2016.018747 10.5014/ajot.2016.018747 26709425

[CR25] Dominick, K. C., Andrews, H. F., Kaufmann, W. E., Berry-Kravis, E., & Erickson, C. A. (2021). Psychotropic drug treatment patterns in persons with fragile X syndrome. *Journal of Child and Adolescent Psychopharmacology,**11*(10), 659–669. 10.1089/cap.2021.0042 10.1089/cap.2021.0042 34818076

[CR26] Eckert, E. M., Dominick, K. C., Pedapati, E. V., Wink, L. K., Shaffer, R. C., Andrews, H., Choo, T. H., Chen, C., Kaufmann, W. E., Tartaglia, N., Berry-Kravis, E. M., & Erickson, C. A. (2019). Pharmacologic interventions for irritability, aggression, agitation, and self-injurious behavior in fragile X syndrome: An initial cross-sectional analysis. *Journal of Autism and Developmental Disorders,**49*(11), 4595–4602. 10.1007/s10803-019-04173-z 31468273 10.1007/s10803-019-04173-z PMC8211361

[CR27] Erickson, C. (2021, February). *Medications for individuals with fragile X syndrome*. National Fragile X Foundation. https://www.fragilex.org/our-research/treatment-recommendations

[CR28] Ethridge, L. E., White, S. P., Mosconi, M. W., Wang, J., Pedapati, E. V., Erickson, C. A., & Sweeney, J. A. (2017). Neural synchronization deficits linked to cortical hyper-excitability and auditory hypersensitivity in fragile X syndrome. *Molecular Autism,**8*(1), 22. 10.1186/s13229-017-0140-1 28596820 10.1186/s13229-017-0140-1 PMC5463459

[CR29] Frankland, P. W., Wang, Y., Rosner, B., Shimizu, T., Balleine, B. W., Dykens, E. M., Ornitz, E. M., & Silva, A. J. (2004). Sensorimotor gating abnormalities in young males with fragile X syndrome and *Fmr1*-knockout mice. *Molecular Psychiatry,**9*, 417–425. 10.1038/sj.mp.4001432 14981523 10.1038/sj.mp.4001432

[CR30] Fung, L. K., & Reiss, A. L. (2016). Moving toward integrative, multidimensional research in modern psychiatry: Lessons learned from fragile X syndrome. *Biological Psychiatry,**80*(2), 100–111. 10.1016/j.biopsych.2015.12.015 26868443 10.1016/j.biopsych.2015.12.015 PMC4912939

[CR31] Galiana-Simal, A., Vela-Romero, M., Romero-Vela, V. M., Oliver-Tercero, N., García-Olmo, V., Benito-Castellanos, P. J., Munoz-Martinez, V., & Beato-Fernandez, L. (2020). Sensory processing disorder: Key points of a frequent alteration in neurodevelopmental disorders. *Cogent Medicine,**7*(1), 1736829. 10.1080/2331205X.2020.1736829

[CR32] Gross, C., Hoffmann, A., Bassell, G. J., & Berry-Kravis, E. M. (2015). Therapeutic strategies in fragile X syndrome: From bench to bedside and back. *Neurotherapeutics,**12*, 584–608. 10.1007/s13311-015-0355-9 25986746 10.1007/s13311-015-0355-9 PMC4489963

[CR33] Hagerman, R. J., Amiri, K., & Cronister, A. (1991). Fragile X checklist. *American Journal of Medical Genetics,**38*(2–3), 283–287. 10.1002/ajmg.1320380223 2018072 10.1002/ajmg.1320380223

[CR34] Hagerman, R. J., Berry-Kravis, E., Kaufmann, W. E., Ono, M. Y., Tartaglia, N., Lachiewicz, A., Kronk, R., Delahunty, C., Hessl, D., Visootsak, J., Picker, J., Gane, L., & Tranfaglia, M. (2009). Advances in the treatment of fragile X syndrome. *Pediatrics,**123*(1), 378–390. 10.1542/peds.2008-0317 19117905 10.1542/peds.2008-0317 PMC2888470

[CR35] Hagerman, R. J., & Hagerman, P. J. (2002). *Fragile X syndrome: Diagnosis, treatment and research* (3rd ed.). The Johns Hopkins University Press.

[CR36] Hagerman, R. J., Hull, C. E., Safanda, J. F., Carpenter, I., Staley, L. W., O’Connor, R. A., Seydel, C., Mazzocco, M. M. M., Snow, K., Thibodeau, S. N., Kuhl, D., Nelson, D. L., Caskey, C. T., & Taylor, A. K. (1994). High functioning fragile X males: Demonstration of an unmethylated fully expanded FMR-1 mutation associated with protein expression. *American Journal of Medical Genetics,**51*, 298–308. 10.1002/ajmg.1320510404 7942991 10.1002/ajmg.1320510404

[CR37] Hagerman, R. J., Jackson, A. W., III., Levitas, A., Rimland, B., Braden, M., Opitz, J. M., & Reynolds, J. F. (1986). An analysis of autism in fifty males with the fragile X syndrome. *American Journal of Medical Genetics Part A,**23*(1–2), 359–374. 10.1002/ajmg.1320230128 10.1002/ajmg.1320230128 3953654

[CR38] Hagerman, R. J., Riddle, J. E., Roberts, L. S., Breese, K., & Fulton, M. (1995). Survey of the efficacy of clonidine in fragile X syndrome. *Developmental Brain Dysfunction,**8*, 336–344.

[CR39] Hall, S. S., Lightbody, A. A., McCarthy, B. E., Parker, K. J., & Reiss, A. L. (2012). Effects of intranasal oxytocin on social anxiety in males with fragile X syndrome. *Psychoneuroendocrinology,**37*(4), 509–518. 10.1016/j.psyneuen.2011.07.020 21862226 10.1016/j.psyneuen.2011.07.020 PMC3353652

[CR40] Heald, M., Adams, D., & Oliver, C. (2020). Profiles of atypical sensory processing in Angelman, Cornelia de Lange and fragile X syndromes. *Journal of Intellectual Disability Research,**64*(2), 117–130. 10.1111/jir.12702 31828905 10.1111/jir.12702

[CR41] Heilman, K. J., Harden, E. R., Zageris, D. M., Berry-Kravis, E., & Porges, S. W. (2011). Autonomic regulation in fragile X syndrome. *Developmental Psychobiology,**53*(8), 785–795. 10.1002/dev.20551 21547900 10.1002/dev.20551 PMC3206129

[CR42] Hersh, J. H., Saul, R. A., & Committee on Genetics. (2011). Health supervision for children with fragile X syndrome. *Pediatrics,**127*(5), 994–1006. 10.1542/peds.2010-3500 21518720 10.1542/peds.2010-3500

[CR43] Hessl, D., Berry-Kravis, E., Cordeiro, L., Yuhas, J., Ornitz, E. M., Campbell, A., Chruscinski, E., Hervey, C., Long, J. M., & Hagerman, R. J. (2009). Prepulse inhibition in fragile X syndrome: Feasibility, reliability, and implications for treatment. *American Journal of Medical Genetics Part B,**150B*(4), 545–553. 10.1002/ajmg.b.30858 10.1002/ajmg.b.30858 PMC2693303 18785205

[CR44] Hessl, D., Glaser, B., Dyer-Friedman, J., Blasey, C., Hastie, T., Gunnar, M., & Reiss, A. L. (2002). Cortisol and behavior in fragile X syndrome. *Psychoneuroendocrinology,**27*(7), 855–872. 10.1016/S0306-4530(01)00087-7 12183220 10.1016/s0306-4530(01)00087-7

[CR45] Hogan, A., Hunt, E., Smith, K., Black, C., Bangert, K., Klusek, J., & Roberts, J. (2021). Trajectories of heart activity across infancy to early childhood differentially predict autism and anxiety symptoms in fragile X syndrome. *Frontiers in Psychiatry,**12*, 727559. 10.3389/fpsyt.2021.727559 34690833 10.3389/fpsyt.2021.727559 PMC8526850

[CR46] Hutt, C., Hutt, S. J., Lee, D., & Ounsted, C. (1964). Arousal and childhood autism. *Nature,**204*, 908–909. 10.1038/204908a0 14235732 10.1038/204908a0

[CR47] Hyde, J., & Garcia-Rill, E. (2019). Autism and arousal. In E. Garcia-Rill (Ed.), *Arousal in neurological and psychiatric diseases* (pp. 83–114). Elsevier.

[CR48] Jirikowic, T. L., Thorne, J. C., McLaughlin, S. A., Waddington, T., Lee, A. K. C., & Hemingway, S. J. A. (2020). Prevalence and patterns of sensory processing behaviors in a large clinical sample of children with prenatal alcohol exposure. *Research in Developmental Disabilities,**100*, 103617. 10.1016/j.ridd.2020.103617 32203885 10.1016/j.ridd.2020.103617

[CR49] Kaufmann, W. E., Kidd, S. A., Andrews, H. F., Budimirovic, D. B., Esler, A., Haas-Givler, B., Stackhouse, T., Riley, C., Peacock, G., Sherman, S. L., Brown, W. T., & Berry-Kravis, B. (2017). Autism spectrum disorder in fragile X syndrome: Cooccurring conditions and current treatment. *Pediatrics,**139*(s3), s194–s206. 10.1542/peds.2016-1159F 28814540 10.1542/peds.2016-1159F PMC5619699

[CR50] Kaufmann, W. E., Raspa, M., Bann, C. M., Gable, J. M., Harris, H. K., Budimirovic, D. B., Lozano, R., FORWARD Consortium. (2022). Latent class analysis identifies distinctive behavioral subtypes in children with fragile X syndrome. *Journal of Autism and Developmental Disorders*. 10.1007/s10803-022-05821-7 36441429 10.1007/s10803-022-05821-7 PMC10258834

[CR51] Kidd, S. A., Lachiewicz, A., Barbouth, D., Blitz, R. K., Delahunty, C., McBrien, D., Visootsak, J., & Berry-Kravis, E. (2014). Fragile X syndrome: A review of associated medical problems. *Pediatrics,**134*(5), 995–1005. 10.1542/peds.2013-4301 25287458 10.1542/peds.2013-4301

[CR52] Klusek, J., Martin, G. E., & Losh, M. (2013). Physiological arousal in autism and fragile X syndrome: Group comparisons and links with pragmatic language. *American Journal on Intellectual and Developmental Disabilities,**118*(6), 475–495. 10.1352/1944.7558-118.6.475 24432860 10.1352/1944.7558-118.6.475 PMC3928802

[CR53] Klusek, J., Moser, C., Schmidt, J., Abbeduto, L., & Roberts, J. (2020). A novel eye-tracking paradigm for indexing social avoidance-related behavior in fragile X syndrome. *American Journal of Medical Genetics Part b: Neuropsychiatric Genetics,**183*(1), 5–16. 10.1002/ajmg.b.32757 31418535 10.1002/ajmg.b.32757 PMC6898737

[CR54] Klusek, J., Roberts, J. E., & Losh, M. (2015). Cardiac autonomic regulation in autism and fragile X syndrome: A review. *Psychological Bulletin,**141*(1), 141–175. 10.1037/a0038237 25420222 10.1037/a0038237 PMC4293203

[CR55] Kolacz, J., Raspa, M., Heilman, K. J., & Porges, S. W. (2018). Evaluating sensory processing in fragile X syndrome: Psychometric analysis of the brain body center sensory scales. *Journal of Autism and Developmental Disorders,**48*, 2187–2202. 10.1007/s10803-018-3491-3 29417435 10.1007/s10803-018-3491-3 PMC9208025

[CR56] Lane, S. J., Mailloux, Z., Schoen, S., Bundy, A., May-Benson, T. A., Parham, L. D., Smith, R. S., & Schaaf, R. C. (2019). Neural foundations of Ayres Sensory Integration^®^. *Brain Sciences,**9*(7), 153. 10.3390/brainsci9070153 31261689 10.3390/brainsci9070153 PMC6680650

[CR57] Little, L. M., Dean, E., Tomchek, S., & Dunn, W. (2018). Sensory processing patterns in autism, attention deficit hyperactivity disorder, and typical development. *Physical and Occupational Therapy in Pediatrics,**38*(3), 243–254. 10.1080/01942638.2017.1390809 29240517 10.1080/01942638.2017.1390809

[CR58] Lyons-Warren, A. M., McCormack, M. C., & Holder, J. L., Jr. (2022). Sensory processing phenotypes in Phelan–McDermid syndrome and *SYNGAP1*-related intellectual disability. *Brain Sciences,**12*(2), 137. 10.3390/brainsci12020137 35203901 10.3390/brainsci12020137 PMC8869824

[CR59] Mayes, L. C. (2000). A developmental perspective on the regulation of arousal states. *Seminars in Perinatology,**24*(4), 267–279. 10.1053/sper.2000.9121 10975433 10.1053/sper.2000.9121

[CR60] McCormick, C., Hepburn, S., Young, G. S., & Rogers, S. J. (2016). Sensory symptoms in children with autism spectrum disorder, other developmental disorders and typical development: A longitudinal study. *Autism,**20*(5), 572–579. 10.1177/1362361315599755 26395236 10.1177/1362361315599755 PMC4918912

[CR61] Miller, L. J., McIntosh, D. N., McGrath, J., Shyu, V., Lampe, M., Taylor, A. K., Tassone, F., Neitzel, K., Stackhouse, T., & Hagerman, R. (1999). Electrodermal responses to sensory stimuli in individuals with fragile X syndrome: A preliminary report. *American Journal of Medical Genetics,**83*(4), 268–279. 10208160

[CR62] Neklyudova, A., Smirnov, K., Rebreikina, A., Martynova, O., & Sysoeva, O. (2022). Electrophysiological and behavioral evidence for hyper- and hyposensitivity in rare genetic syndromes associated with autism. *Genes,**13*(4), 671. 10.3390/genes13040671 35456477 10.3390/genes13040671 PMC9027402

[CR63] Pieretti, M., Zhang, F. P., Fu, Y.-H., Warren, S. T., Oostra, B. A., Caskey, C. T., & Nelson, D. L. (1991). Absence of expression of the *FMR-1* gene in fragile X syndrome. *Cell,**66*(4), 817–822. 10.1016/0092-8674(91)90125-I 1878973 10.1016/0092-8674(91)90125-i

[CR64] Rais, M., Binder, D. K., Razak, K. A., & Ethell, I. M. (2018). Sensory processing phenotypes in fragile X syndrome. *ASN Neuro,**10*, 1–19. 10.1177/1759091418801092 10.1177/1759091418801092 PMC6149018 30231625

[CR65] Raspa, M., Wheeler, A., Okoniewski, K. C., Edwards, A., & Scott, S. (2023). Research gaps in fragile X syndrome: An updated literature review to inform clinical public health practice. *Journal of Developmental and Behavioral Pediatrics,**44*(1), e56–e65. 10.1097/DBP.0000000000001134 36219479 10.1097/DBP.0000000000001134 PMC9770151

[CR66] Raspa, M., Wylie, A., Wheeler, A. C., Kolacz, J., Edwards, A., Heilman, K., & Porges, S. W. (2018). Sensory difficulties in children with a *FMR1* premutation. *Frontiers in Genetics,**9*, 351. 10.3389/fgene.2018.00351 30233641 10.3389/fgene.2018.00351 PMC6127619

[CR67] Roberts, J. E., Boccia, M. L., Bailey, D. B., Jr., Hatton, D. D., & Skinner, M. (2001). Cardiovascular indices of physiological arousal in boys with fragile X syndrome. *Developmental Psychobiology.,**39*(2), 107–123. 10.1002/dev.1035 11568881 10.1002/dev.1035

[CR68] Rojas, D. C., Benkers, T. L., Rogers, S. J., Teale, P. D., Reite, M. L., & Hagerman, R. J. (2001). Auditory evoked magnetic fields in adults with fragile X syndrome. *NeuroReport,**12*(11), 2573–2576. 10.1097/00001756-200108080-00056 11496151 10.1097/00001756-200108080-00056

[CR69] Rotschafer, S. E., & Razak, K. A. (2014). Auditory processing in fragile X syndrome. *Frontiers in Cellular Neuroscience,**8*, 19. 10.3389/fncel.2014.00019 24550778 10.3389/fncel.2014.00019 PMC3912505

[CR70] Schaaf, R. C., Benevides, T., Mailloux, Z., Faller, P., Hunt, J., van Hooydonk, E., Freeman, R., Leiby, B., Sendecki, J., & Kelly, D. (2014). An intervention for sensory difficulties in children with autism: A randomized trial. *Journal of Autism and Developmental Disorders,**44*, 1493–1506. 10.1007/s10803-013-1983-8 24214165 10.1007/s10803-013-1983-8 PMC4057638

[CR71] Schaaf, R. C., Dumont, R. L., Arbesman, M., & May-Benson, T. A. (2018). Efficacy of occupational therapy using Ayres sensory integration^®^: A systematic review. *The American Journal of Occupational Therapy,**72*(1), 7201190010p1-7201190010p10. 10.5014/ajot.2018.028431 29280711 10.5014/ajot.2018.028431

[CR72] Sherman, S. L., Kidd, S. A., Riley, C., Berry-Kravis, E., Andrews, H. F., Miller, R. M., Lincoln, S., Swanson, M., Kaufmann, W. E., & Brown, W. T. (2017). FORWARD: A registry and longitudinal clinical database to study fragile X syndrome. *Pediatrics,**139*(Suppl 3), S183–S193. 10.1542/peds.2016-1159E 28814539 10.1542/peds.2016-1159E PMC5621599

[CR73] Shoen, S. A., Lane, S. J., Mailloux, Z., May-Benson, T., Parham, L. D., Smith Roley, S., & Schaaf, R. C. (2019a). A systematic review of Ayres sensory integration for children with autism. *Autism Research,**12*(1), 6–19. 10.1002/aur.2046 30548827 10.1002/aur.2046 PMC6590432

[CR74] Shoen, S. A., Lane, S. J., Schaaf, R. C., Mailloux, Z., Parham, L. D., Roley, S. S., & May-Benson, T. (2019b). Ayres sensory integration meets criteria for an evidence-based practice: A response to Stevenson. *Autism Research,**12*(8), 1154–1155. 10.1002/aur.2164 31254316 10.1002/aur.2164

[CR75] Smith Roley, S. S., Blanche, E. I., & Schaaf, R. C. (2001). *Understanding the nature of sensory integration with diverse populations*. Pro-ed.

[CR76] Stackhouse, T. M., Scharfenaker, S. K., Lachiewicz, A. M., Burgess, D., Hessl. D., Blitz, R., Burgess, K., Rohlik, D., Griess Hess L., Kidd, S. A., & Berry-Kravis, E. (2014, May). *Sensory processing and integration issues in individuals with fragile X syndrome*. National Fragile X Foundation. https://www.fragilex.org/our-research/treatment-recommendations

[CR77] Taylor, A. K., Safanda, J. F., Fall, M. Z., Quince, C., Lang, K. A., Hull, C. E., Carpenter, I., Staley, L. W., & Hagerman, R. J. (1994). Molecular predictors of cognitive involvement in female carriers of fragile X syndrome. *JAMA,**271*(7), 507–514. 10.1001/jama.1994.03510310037035 8301764

[CR78] Tomchek, S. D., & Dunn, W. (2007). Sensory processing in children with and without autism: A comparative study using the short sensory profile. *American Journal of Occupational Therapy,**61*(2), 190–200. 10.5014/ajot.61.2.190 10.5014/ajot.61.2.190 17436841

[CR79] Tranfaglia, M. R. (2011). The psychiatric presentation of fragile X: Evolution of the diagnosis and treatment of the psychiatric comorbidities of fragile X syndrome. *Developmental Neuroscience,**33*, 337–348. 10.1159/000329421 21893938 10.1159/000329421

[CR80] Tsiouris, J. A., & Brown, W. T. (2004). Neuropsychiatric symptoms of fragile X syndrome: Pathophysiology and pharmacotherapy. *CNS Drugs,**18*(11), 687–703. 10.2165/00023210-200418110-00001 15330685 10.2165/00023210-200418110-00001

[CR81] Watson, C., Hoeft, F., Garrett, A. S., Hall, S. S., & Reiss, A. L. (2008). Aberrant brain activation during gaze processing in boys with fragile X syndrome. *Archives of General Psychiatry,**65*(11), 1315–1323. 10.1001/archpsyc.65.11.1315 18981343 10.1001/archpsyc.65.11.1315 PMC4445973

[CR82] Wheeler, A. C., Raspa, M., Bishop, E., & Bailey, D. B. (2016). Aggression in fragile X syndrome. *Journal of Intellectual Disability Research,**60*(2), 113–125. 10.1111/jir.12238 26628097 10.1111/jir.12238

[CR83] Wilbarger, P., & Wilbarger, J. (1991). *Sensory defensiveness in children aged 2–12: An intervention guide for parents and other caretakers*. Avanti Educational Programs.

[CR84] Willemsen, R., Levenga, J., & Oostra, B. A. (2011). CGG repeat in the *FMR1* gene: Size matters. *Clinical Genetics,**80*(3), 214–225. 10.1111/j.1399-0004.2011.01723.x 21651511 10.1111/j.1399-0004.2011.01723.x PMC3151325

[CR85] Zafarullah, M., & Tassone, F. (2019). Molecular biomarkers in fragile X syndrome. *Brain Sciences,**9*(5), 96. 10.3390/brainsci9050096 31035599 10.3390/brainsci9050096 PMC6562871

[CR86] Zimmer, M., Desch, L., Rosen, L. D., Bailey, M. L., Becker, D., Culbert, T. P., McClafferty, H., Sahler, O. J. Z., Vohra, S., Liptak, G. S., Adams, R. C., Burke, R. T., Friedman, S. L., Houtrow, A. J., Kalichman, M. A., Kuo, D. Z., Levy, S. E., Norwood, K. W., Turchi, R. M., & Wiley, S. E. (2012). Sensory integration therapies for children with developmental and behavioral disorders. *Pediatrics,**129*(6), 1186–1189. 10.1542/peds.2012-0876 22641765 10.1542/peds.2012-0876

